# GTPase domain driven dimerization of SEPT7 is dispensable for the critical role of septins in fibroblast cytokinesis

**DOI:** 10.1038/srep20007

**Published:** 2016-01-28

**Authors:** Megha Abbey, Cosima Hakim, Roopsee Anand, Juri Lafera, Axel Schambach, Andreas Kispert, Manuel H. Taft, Volkhard Kaever, Alexey Kotlyarov, Matthias Gaestel, Manoj B. Menon

**Affiliations:** 1 Institute of Physiological Chemistry, Hannover Medical School, Hannover- 30625, Germany; 2 Institute of Biophysical Chemistry, Hannover Medical School, Hannover- 30625, Germany; 3 Institute of Experimental Hematology, Hannover Medical School, Hannover- 30625, Germany; 4 Institute of Molecular Biology, Hannover Medical School, Hannover- 30625, Germany; 5 Institute of Pharmacology, Hannover Medical School, Hannover- 30625, Germany; 6 Research Core Unit Metabolomics, Hannover Medical School, Hannover- 30625, Germany

## Abstract

Septin 7 (SEPT7) has been described to be essential for successful completion of cytokinesis in mouse fibroblasts, and *Sept7*-deficiency in fibroblasts constitutively results in multinucleated cells which stop proliferation. Using *Sept7*^*flox/flox*^fibroblasts we generated a cellular system, where the cytokinetic defects of Cre-mediated deletion of the *Sept7* gene can be rescued by ectopically expressed doxycycline-inducible wild type SEPT7. Using this system, we analyzed the ability of SEPT7-mutants with alterations in their GTPase domain-dependent dimerization to prevent multinucleation and rescue proliferation. Although biochemical analysis of the mutants demonstrates differences in homo- and/or hetero-polymerization, in GTP-binding and/or GTPase activities, all analyzed mutants were able to rescue the cytokinesis phenotype of *Sept7*^*flox/flox*^fibroblasts associated with Cre-mediated deletion of endogenous *Sept7*. These findings indicate that the ability of septins to assemble into well-defined SEPT7-dimerization dependent native filaments is dispensable for cytokinesis in fibroblasts and opens the way to search for other mechanisms of the involvement of SEPT7 in cytokinesis.

Septins are an evolutionarily conserved class of GTP-binding cytoskeletal proteins with unique properties and diverse functions. They are conserved from yeast to humans, and most eukaryotes except plants and some algae harbour more than one septin gene in their genome. Initially discovered as factors critical for budding in the yeast *Saccharomyces cerevisiae*, septins are gaining prominence as the fourth component of the eukaryotic cytoskeleton^1^,^2^. Septins are classified under the translation factor (TRAFAC) class of P-loop GTP-binding proteins, possess a central GTPase domain similar to the Ras family of small GTPases, and display strong guanine nucleotide binding and slow GTP-hydrolysis activity *in vitro*^3^,^4^.

Mice and humans possess 13 septin genes which are classified into 4 different groups (SEPT2, SEPT3, SEPT6 and SEPT7) based on phylogenetic analysis^1^,^5^. The unique feature of the septins is their capability to heteropolymerise and form a diverse array of higher order structures which include filaments, gauges and rings. Biochemical purification and structural analysis of a human SEPT2-SEPT6-SEPT7 trimeric complex established the basic rules governing the non-polar assembly of septin heteropolymers which also confirmed the importance of the GTPase domain of septins in filament formation^6^. In a dual approach coupling X-ray crystallographic analysis with electron microscopy, it was established that the filaments are formed with alternating N-C interfaces (formed by the interaction of N- and C- termini of the septin subunits) and G-G interfaces (formed by the interaction of the GTPase domains) between the subunits. The basic repeat unit consisted of a hexamer- SEPT7:6:2:2:6:7 where SEPT2-SEPT2 and SEPT6-SEPT7 interactions occurred via N-C interfaces and SEPT2-SEPT6 and SEPT7-SEPT7 interactions occurred via the G-G interface^6^. While SEPT2 and SEPT7 were associated with GDP in the hexamer, SEPT6 was GTP-bound, leading to the conclusion that SEPT6 family proteins are GTPase-deficient^6^,^7^. Later studies established the presence of an octameric septin assembly with SEPT9 associated at the terminal positions of the canonical hexamer^8^. Higher order assembly of septin filaments are facilitated by the association of these oligomers to the cell membrane^9^. These biophysical studies proposed a model where the position occupied by each septin subunit can be replaced by other group members. As SEPT7 is the only member of its group, it is an essential component in the canonical septin assembly^6^,^10^,^11^.

Although mammalian septins have been implicated in many processes including neuronal morphogenesis, platelet-degranulation, macrophage phagocytosis or calcium signalling^1^,^2^,^5^, the evolutionarily most conserved role of septins is in cytokinesis, the final step of cell divison^12^,^13^,^14^,^15^,^16^. While the formation of septin filaments has been found to be a prerequisite for efficient budding in yeast^17^,^18^, the precise role of septin filament formation in mammalian cytokinesis has remained unknown. We recently generated *Sept7* conditional knockout mice and demonstrated the obligate requirement of the gene in embryonic development and fibroblast cytokinesis^14^. Consistent with the pivotal role of SEPT7 in septin assembly, the deletion of *Sept7* in fibroblasts was associated with co-depletion of other core septin subunits. In the present study, we utilized *Sept7-*deficient fibroblasts to generate a rescue model for analysis of the effect of different GTPase domain mutations of SEPT7 in fibroblast cytokinesis. Here, we show that neither native septin filaments nor the G-G homodimerization interface of SEPT7 is necessary for SEPT7-dependent completion of fibroblast cytokinesis.

## Results

### Generation of a doxycycline-inducible rescue system for monitoring septin-dependent cytokinesis

**Figure 1:**
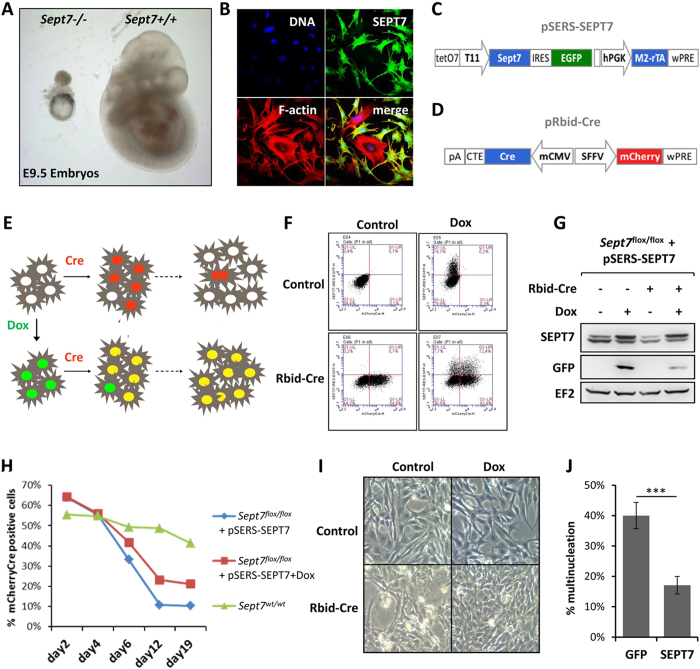
A rescue model to investigate the obligate role of SEPT7 in cytokinesis. (**A)** Embryonic day 9.5 embryos showing strong growth defect on *Sept7* deletion. (**B)** Cre transduction induced deletion of Sept7 in immortalized fibroblasts leads to obligate multinucleation. SEPT7-immunostaining, phalloidin staining for F-actin, DAPI for DNA are shown. (**C,D)** Expression cassette and important features in the bi-cistronic SEPT7 expression vector (**C**) and the bidirectional Cre-mCherry expression vector (**D**) used in the study. (**E)** Schematic representation for the basis of the dual colour flow-cytometric assay for SEPT7 rescue model. (**F**) Flow-cytometric analysis of double transduced MEFs showing mCherry and dox-inducible GFP signals. (**G)** Immunoblot analysis showing the Cre-mediated depletion and doxycycline-induced rescue of SEPT7 expression in MEFs. (**H)** Flow-cytometric analysis for the percentage of mCherry Cre positive cells at the indicated time-points post transduction, in the presence or absence of doxycycline. Wild-type cells transduced with mCherry Cre is shown as control. (**I)** Representative microscopic images showing the effect of Dox-treatment on Cre-induced multinucleation in double-transduced MEFs. (**J)** *pSERS-SEPT7-IRES-GFP* or *pSERS-GFP* expressing cells were transduced with mCherry Cre and were sorted for mCherry/GFP-double positive cells. The cells were analyzed for multinucleation after SYTOX green staining (student’s t-test, n = 4,*** denotes a p value of 0.00025).

It has previously been shown that deletion of three members of the septin gene family, namely *Sept7, Sept9 and Sept11* results in embryonic lethality. *Sept11*^*−/−*^ embryos die between embryonic day (E)11.5–13.5^19^, *Sept9*^*−/−*^ embryos at E10^20^ while *Sept7*-deletion leads to lethality between E7-10.5^14^. Reinspection of litters at E9.5 showed that *Sept7*-mutant embryos retained egg cylinder morphology at this stage indicating that they did not gastrulate as their wildtype littermates (Fig. 1A). Interestingly, in contrast to the mild phenotypes associated with the deletion of *Sept9* and *Sept11* in fibroblast cultures^19^,^20^, it was not possible to maintain and characterize *Sept7-*deficient fibroblasts in culture since they failed to proliferate^14^. Our previous studies demonstrated an absolute requirement of *Sept7* in fibroblast cytokinesis and cell proliferation. Genetic deletion of *Sept7* leads to destabilization of the core septin cytoskeleton resulting in obligate multinucleation of adult and embryonic fibroblasts (Fig. 1B)^14^. To further investigate the role of septins in cytokinesis, we generated a doxycycline (dox)-inducible SEPT7-rescue model. For this purpose, fibroblasts isolated from *Sept7*^*flox/flox*^ mice were transduced with a retroviral vector harboring a dox-inducible SEPT7 expression cassette with IRES-driven expression of GFP from the same transcript (pSERS-SEPT7, Fig. 1C). These cells were further transduced with another retrovirus expressing mCherry and Cre from a bidirectional constitutive promoter (pRbid-Cre, Fig. 1D)^14^. In the double transduced cells, Cre-expression leads to the deletion of the endogenous *Sept7* allele and these knockout cells could be specifically monitored by mCherry fluorescence. Consistent with the inability of *Sept7* KO cells to proliferate, the mCherry positive cells should gradually disappear from culture in the absence of doxycycline. Inducible expression of exogenous SEPT7 should significantly stabilize the proportion of mCherry positive cells in culture and prevent the formation of large multinucleated cells on Cre-transduction as schematically represented in Fig. 1E. We monitored GFP and mCherry expression in the transduced cells in the presence and absence of doxycycline by FACS (Fig. 1F) and GFP and SEPT7 expression by Western blot (Fig. 1G). The impaired proliferation of Cre expressing *Sept7*^*flox/flox*^ fibroblasts is significantly rescued by doxycycline-induced expression of SEPT7 (Fig. 1H). Indeed, Cre expression leads to multinucleation, which is prevented by parallel induction of SEPT7 by doxycycline as seen by light microscopy (Fig. 1I). To further verify, that multinucleation is indeed the reason for the depletion of mCherry positive cells from the population, we sorted mCherry/GFP double positive populations from cells expressing SEPT7 (IRES-GFP) or empty vector (GFP). Consistent with the other results, microscopic analysis revealed significantly higher proportion of multinucleation in the empty vector transduced cells (Fig. 1J & Supplementary Fig. 1).

### Generation and characterization of SEPT7-mutants affecting GTPase-domain dependent dimerization

**Figure 2:**
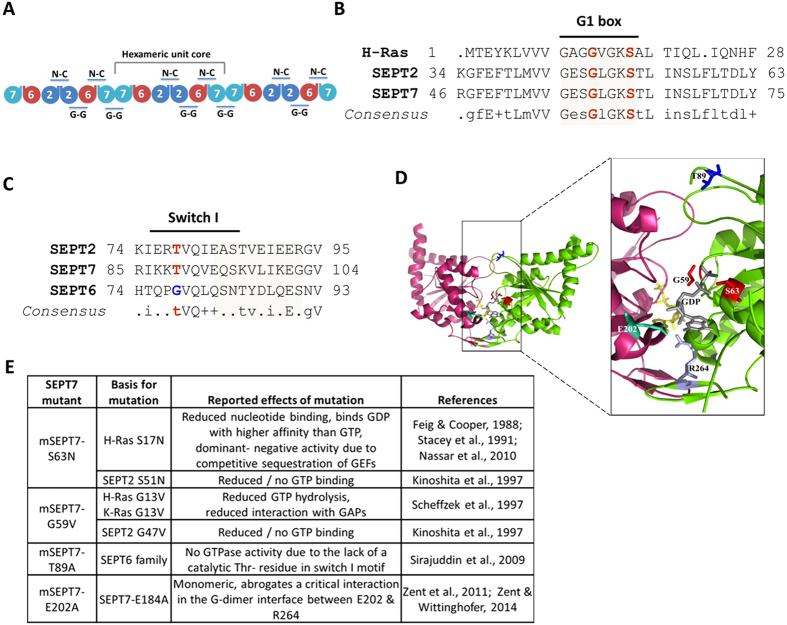
GTPase domain mutants of SEPT7. **(A)** Scheme for the organization of a septin hetero-oligomeric complex depicting the important interfaces of interaction. **(B)** Alignment of human H-Ras (NP_001123914), mouse Sept2 (NP_001153189) and SEPT7 (NP_001192296) sequences showing conserved regions in the GTPase domain- G1 box highlighted**. (C)** Sequence comparison of GTPase domain – switch I region residues in mouse SEPT7, SEPT2 and SEPT6 (NP_001170795.1). The conserved Threonine (T) residue critical for GTP binding and hydrolysis (in red) is absent in SEPT6 which makes it catalytically inactive. The critical residues selected for mutagenesis are in bold. (**D)** The structure of a homodimer of human SEPT7 bound to GDP (PDB 3T5D). The corresponding residues chosen for mutation in mouse SEPT7 are labeled and represented in the form of colored sticks [E202 in Chain A (magenta) and other sites indicated in chain B (green)] (Represented using PyMOL, loops in the structure were smoothened for a better representation). (**E)** Mutations introduced in SEPT7, expected outcomes and the scientific basis for the mutations tabulated.

SEPT7 is a pivotal member of the septin family and is critical for the formation of septin filaments. The importance of SEPT7 in septin filament assembly is well supported by the absolute requirement of this protein for fibroblast cytokinesis and the co-depletion of the core-septin components SEPT6 and SEPT2 upon SEPT7 deletion^14^. The most well characterized core-unit of the septin structure is a hexamer consisting of SEPT7:6:2:2:6:7. The higher order polymerization of this hexamer is achieved via the homomeric interaction of the terminal SEPT7 moieties through their GTPase domain interfaces^6^ (Fig. 2A). Hence, the G-domain interface of SEPT7 seems to be important for higher order filament assembly. Moreover, septins are active GTPases and GTP-binding was found to be important for the functions of certain yeast and *Drosophila* septins^4^,^21^,^22^. However, studies on the role of GTPase activity of mammalian septins have been restricted to *in vitro* biochemical analysis so far^7^,^23^,^24^,^25^. We performed homology alignments of SEPT7 GTPase domain with that of SEPT2 and H-Ras and identified critical residues G59 and S63 in the G1 box required for maintaining GTPase activity and GTP-binding^26^,^27^,^28^ (Fig. 2B). In addition, the critical residue T89 was identified by comparisons between SEPT2/SEPT7 and the catalytically inactive GTPase domains of the SEPT6 family^7^ (Fig. 2C). Apart from this, we also analyzed a mutation of SEPT7 (SEPT7 E202A, reported as E184A) outside the conserved GTPase domains, previously reported to affect dimerization via the G-domain^23^,^24^. In the structure of dimerized SEPT7 GTPase domains, Glutamate-202 establishes close contacts with Arginine-264 of the second SEPT7 molecule and the ribose sugar of the attached nucleotide, stabilizing the G-G interaction (Fig. 2D). SEPT7 Mutations in the respective residues were introduced in mouse SEPT7 (isoform2- *NP_001192296.1*- G59V, S63N, T89A and E202A) coding sequence (Fig. 2E) and further characterized.

### Point mutations at E202 and G59 abrogate homo-polymeric assembly of GFP-tagged SEPT7

**Figure 3:**
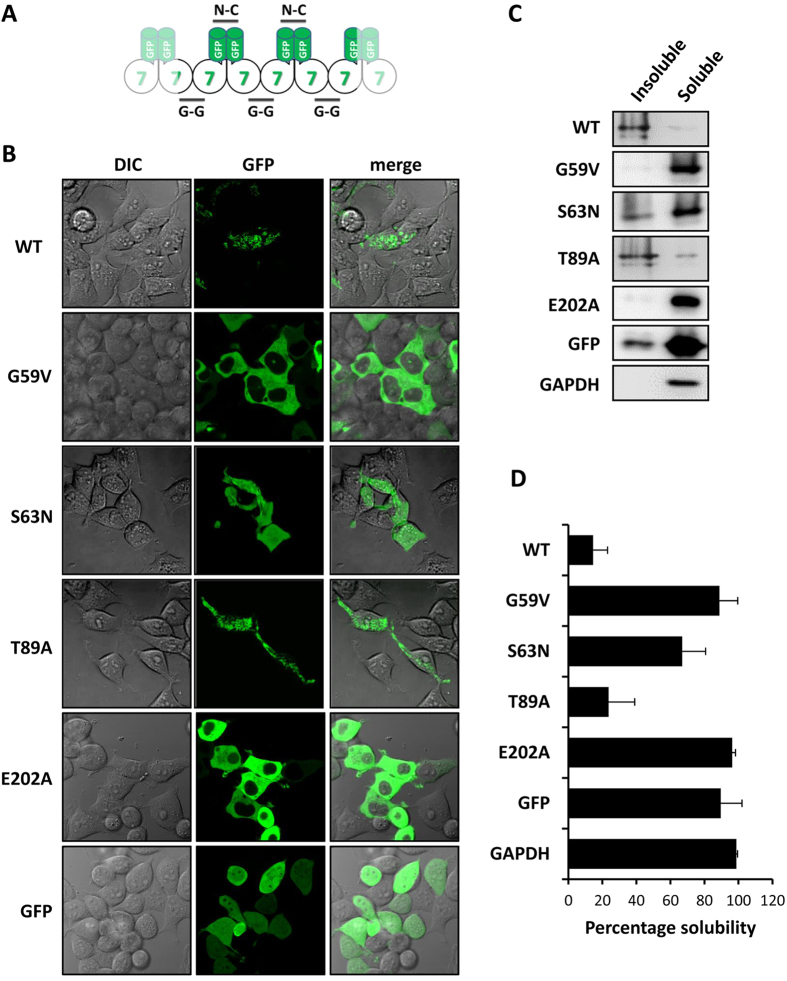
The subcellular distribution and solubility of GFP-tagged SEPT7 mutants in transfected cells. **(A)** Schematic representation of homopolymerization by GFP-SEPT7. (**B)** Live cell imaging showing the structural organization of GFP-tagged wild-type (WT) and mutants SEPT7 in transfected 293T cells. Fluorescent images (GFP) as well as differential interference contrast (DIC) images are shown. (**C)** Triton soluble and insoluble fractions of lysates were probed with anti GFP antibodies to detect GFP-tagged SEPT7 wild-type (WT) and mutants. GFP alone and GAPDH are shown as control. (**D)** Quantified band intensities from (**C**) represented as percentage solubility (average from three independent experiments plotted with standard deviation).

GFP-tagged SEPT7, when over-expressed in mammalian cells, forms filamentous aggregates mostly consisting of homopolymerized septins (Fig. 3A) which are hardly solubilized even by high detergent containing lysis buffers (1% Triton), while endogenous SEPT7 and other core septin isoforms are almost completely solubilized under similar conditions (Supplementary Fig. 2). The homopolymerization is restricted to certain septin family members, such as SEPT7 and SEPT2, while other members (SEPT6) only undergo hetero-polymer assembly which necessitates co-expression of other core septins for filament formation. We first characterized the mutants generated in their ability to homopolymerize when ectopically expressed in HEK293T cells. As expected, wild-type SEPT7 shows the formation of triton-insoluble filaments, while the different mutants showed variable tendencies of filament formation and solubility (Fig. 3B–D). Consistent with the previously published data demonstrating SEPT7-E202A as a monomeric non-dimerizing mutant, this mutant showed completely diffused cellular distribution and high solubility similar to that of GFP. Interestingly, the G59V mutation affecting a conserved residue in the G1 box homologous to Gly-13 in H-Ras and critical for GTP-hydrolysis and GAP interaction resulted in a cellular distribution and solubility profile similar to the E202A mutant. While the solubility of the T89A mutant was more comparable to that of wild-type SEPT7, the S63N mutant showed significantly enhanced solubility. These solubility profiles were consistent with their capacity to form filaments as observed by fluorescence microscopy.

### Novel G-domain mutants of SEPT7 display altered nucleotide hydrolysis kinetics

**Figure 4:**
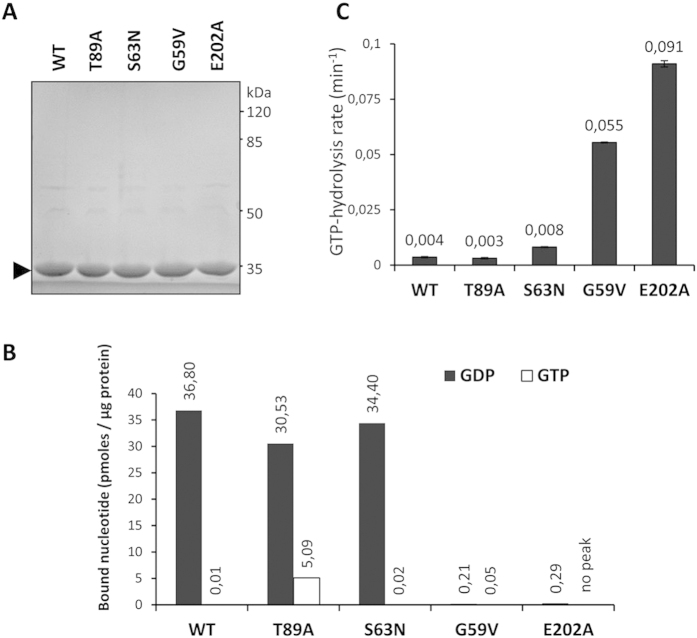
Effect of mutations on the GTPase activity of SEPT7. **(A)** Coomassie stained gel showing equal amounts of pure GST-tag removed preparations of SEPT7 WT and mutants. (**B**) Native guanine nucleotide association of recombinant SEPT7 GTPase domains monitored by mass spectrometry. (**C**) Regenerative coupled-enzyme GTP hydrolysis assays for SEPT7 wild-type (WT), G59V, S63N, T89A and E202A mutants.

Since the mutations of the GTPase domain as well as the previously described E202A mutation hindering G-domain mediated dimerization showed significantly altered homopolymerization properties in over-expressed HEK293T cells, we further monitored the effect of these mutations on the guanine nucleotide binding properties of SEPT7. Higly pure untagged SEPT7-GTPase domains (residues 47 to 315) were prepared by GST-tag affinity purification followed by tag cleavage (Fig. 4A). Equal amount of the purified proteins were used for monitoring guanine nucleotide association by mass spectrometry. As previously reported, SEPT7-GTPase domains purified from bacteria are already preloaded with GDP^23^. In general the G59V and E202A mutant proteins showed highly reduced nucleotide-association with no bound GTP detected with the E202A mutant. These data hint towards reduced nucleotide-binding affinities for these mutants. While there was greater than 4000-fold excess of GDP bound to wild-type GTPase domain compared to GTP, the T89A mutant showed a significantly high proportion of GTP-bound protein (<6 fold GDP/GTP) (Fig. 4B). Interestingly, the T89A mutation was modelled on the basis of the corresponding residue in the SEPT6 family proteins, which renders them GTPase-inactive (Fig. 2E)^24^. As a result of the absence of a critical threonine residue in the switch-I region, SEPT6 is associated with non-hydrolyzed GTP in the crystal structure of the core-hexamer, while SEPT2/SEPT7 have GDP associated with their G-domains^7^. Consistent with this idea, SEPT6-like mutation of SEPT7 seems to generate a protein which is more GTP-associated probably due to the lack of intrinsic GTPase activity.

To further assess the GTP-GDP exchange and GTPase activity of the mutants in a more quantitative way, we performed a quantitative GTP-hydrolysis assay coupled to NADH-dependent recycling of GTP^29^,^30^. This spectrophotometric assay is performed at saturating GTP concentrations, where every free GDP molecule generated is rapidly regenerated to GTP, consuming one molecule of NADH and the hydrolysis rates are quantified as decrease in the absorption of NADH. In accordance with the published data^24^, the wild-type SEPT7 showed very low GTP-turnover rates and the E202A mutation resulted in more than 20 fold enhancement of GTPase activity of the mutant (Fig. 4C). Extreme low rates of GTP-hydrolysis associated with wild-type SEPT7 could be due to the GTP-hydrolysis induced dimerization of the proteins, which could prevent easy dissociation of GDP and hence over all GTP-turnover rates^24^. While the wild-type, S63N and T89A mutants showed low GTPase activities, the G59V mutant exhibited GTP-hydrolysis rates comparable to that of the E202A mutant. This confirms the presence of an inverse relation between GTP-turnover rates of the mutants and their capacity to homodimerize.

### Doxycycline-induced expression of all mutant SEPT7 proteins rescue the proliferation defects associated *Sept7*-deletion

**Figure 5:**
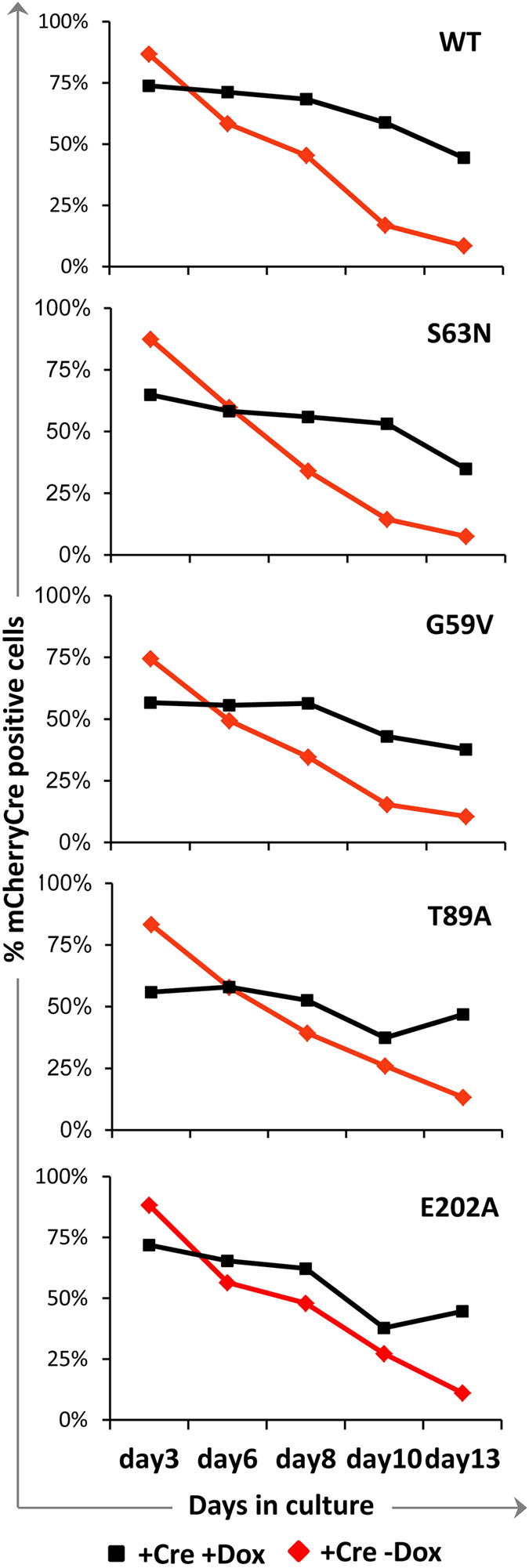
Monitoring the rescue of SEPT7 depletion associated lethality by flow-cytometry. The plots show flow-cytometric quantification of the persistence of mCherry/Cre positive population over time in the presence and absence of doxycycline (Dox). Dox-induced expression of SEPT7 wild-type (WT) or mutants significantly enhance the proportion of mCherry positive cells (refer Fig. 1E), suggesting effective rescue.

**Figure 6:**
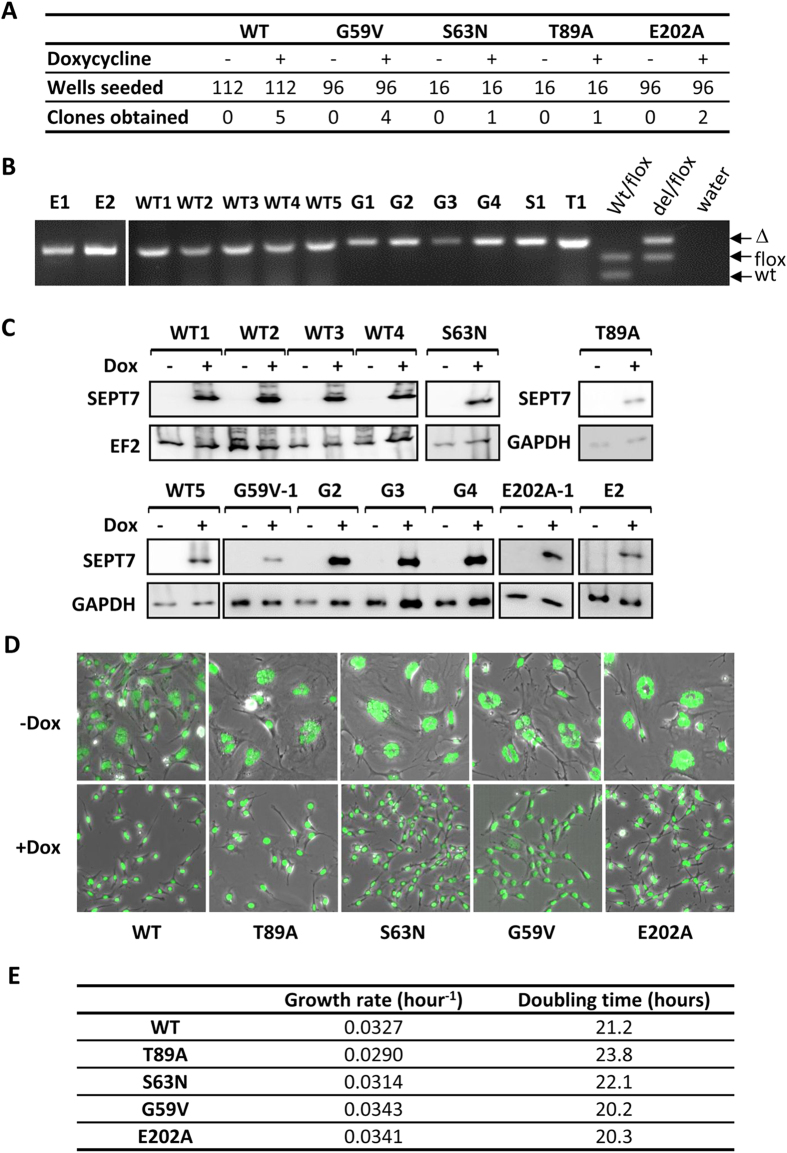
Generation of doxycycline inducible rescue clones of *Sept7* knockout MEFs. **(A)** Rescue clones obtained by single-cell cloning strategy with double transduced MEFs. Indicated numbers of wells were seeded in 96-well plates in the presence or absence of doxycycline and the number of wells which successfully gave actively proliferating cell clones are indicated. (**B–D)** Verification of SEPT7 rescue clones. Genotyping analysis of the rescue clones (**B**), Immunoblot analysis for dox-inducible SEPT7 expression in the rescue clones with additional loading control blots (**C**) and SYTOX green staining for nuclei and microscopic anaylsis of multinucleation in the rescue clones propagated in the presence and absence of doxycycline (**D**). (**E**) Doubling time for the clones were calculated by using WST-1 cell proliferation assay and the results are tabulated.

After the *in vitro* characterization of the mutants, which clearly demonstrated altered biochemical properties, we analyzed whether these mutants could rescue the abscission defect associated with *Sept7*-deficiency. The most interesting candidates were the E202A and G59V mutants, which clearly presented altered polymerization and GTPase activity kinetics when compared to WT. We now utilized the Dox-inducible rescue model where the persistence of the mCherry positive (Cre-positive) cells indicates survival of knockout cells (cf. Fig. 1). Surprisingly, dox-inducible expression of all SEPT7 mutants, including the G59V and E202A mutants, could effectively stabilize the proportion of mCherry-positive cell population, suggesting efficient rescue of the *Sept7*-deficient phenotype (Fig. 5). To further analyze this unexpected observation in more detail, we performed serial dilution and single-cell cloning to isolate single clones of *Sept7*-knockout cells rescued by dox-inducible SEPT7. Genotyping confirmed isolation of single-cell clones with genetic deletion of the *Sept7* gene (exon 4) and, in addition, immunoblotting analysis confirmed Dox-dependent expression of the SEPT7-IRES-EGFP cassette in the clones (Fig. 6A–C). The clones could be continuously maintained in medium supplemented with doxycycline and its withdrawal led to cytokinesis defects resulting in large multinucleated cells consistent with the phenotype of *Sept7*-deficient fibroblasts (Fig. 6D). To quantitatively compare the relative efficiency of the mutant septins to rescue proliferation, we calculated the doubling time of the rescued clones. Surprisingly, all cell lines proliferated at very similar rates regardless whether mutant or WT SEPT7 is expressed (Fig. 6E). Again, these data clearly demonstrate that the various mutants of SEPT7, despite their altered biochemical properties, are able to perform the obligate function of SEPT7 in cytokinesis in the *Sept7*-deficient background.

### Altered septin filament architecture in the mutant SEPT7-rescued fibroblasts

**Figure 7:**
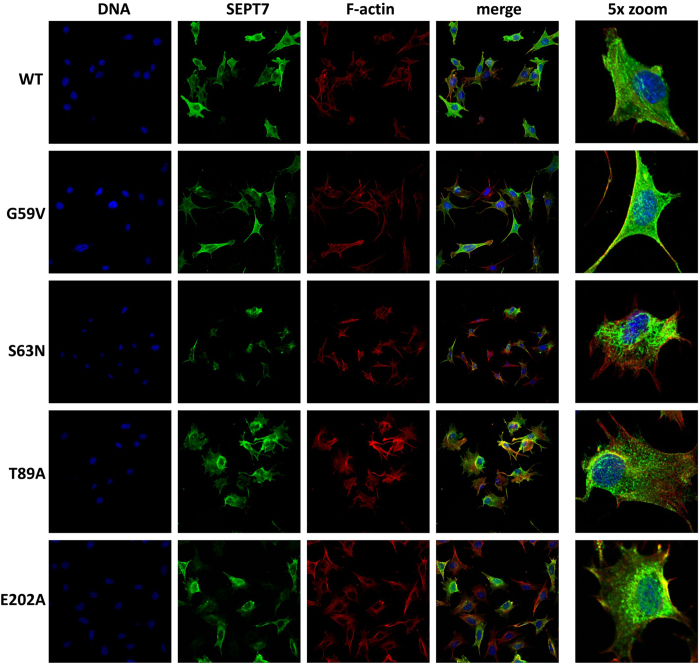
Organisation of SEPT7 containing filaments in the rescued MEF clones. Confocal laser scanning immuno-fluorescence analysis depicting the subcellular distribution of wild-type and mutant SEPT7 in respective rescue clones. The cells were stained for SEPT7, DNA (DAPI) and F-actin (phalloidin).

Physiological septin filaments are hetero-oligomeric complexes consisting of core septin hexamers (SEPT7:6:2:2:6:7) and octamers (9:7:6:2:2:6:7:9), with SEPT7-G domain interactions being critical for further polymerization of the hexameric complexes. Immunofluorescence analysis of the rescued cells showed altered SEPT7 organization in the case of the mutants (Fig. 7). It is interesting to note that the G59V and E202A mutants, that showed completely diffused distribution on overexpression in HEK293T cells, formed some filamentous structures in the rescued cells albeit with visible differences compared to the native wild-type SEPT7 containing filaments. In comparison to the long and slender filaments of SEPT7 wild-type, S63N filaments were long and thick. G59V and E202A mutants appeared as short filamentous structures while T89A mutant formed aggregated foci (punctate structures) diffusely distributed throughout the cell (Fig. 7).

### All SEPT7 mutant rescued MEFs contain soluble SEPT7-containing oligomeric complexes

**Figure 8:**
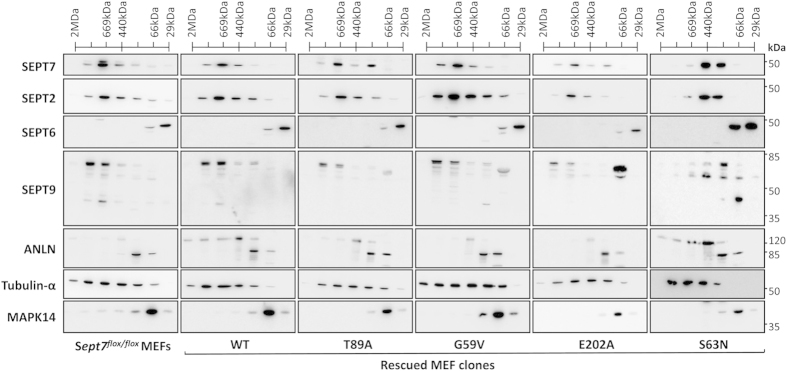
Monitoring the oligomerisation status of septins in the rescued MEF clones. Superose6 10/30 size-exclusion chromatography analysis of doxycycline-induced SEPT7 from the rescue clones. Immunoblot analysis of the fractions obtained from *Sept7*^*flox/flox*^ MEFs and from the dox-induced rescue clones of SEPT7 wild-type (WT), T89A, G59V, E202A and S63N mutants (from left to right).

The presence of higher order structural assembly of the mutants, including the homodimerization deficient E202A mutant, supports the notion that the septin assembly in the rescued fibroblasts is largely heteropolymeric. To monitor the effect of different mutations on the G-domain-dependent interactions in more detail and to specifically analyze the oligomeric organization of the septins in fibroblasts, we performed gel-filtration analysis of triton-solubilized fibroblast lysates. Detergent solubilization coupled to an ultracentrifugation step excludes higher-order septin filaments and aggregates and makes it possible to analyze the oligomer assembly. Initially, we monitored the distribution of the core septin subunits in the *Sept7*-floxed fibroblast line to look at the hetero-oligomerization status of endogenous SEPT7 (Fig. 8, left panel). SEPT7 and SEPT2 signals peaked in fractions in ~600 kDa range suggesting the presence of higher order complexes consisting of septin and non-septin interaction partners. SEPT9 signals were also observed in these fractions, while the peak signals were observed in slightly higher molecular weights. Unexpectedly, SEPT6 signals are detectable in very low molecular weight fractions suggesting that in these cells, SEPT6 is not a part of the oligomeric complexes of septins consisting of SEPT2, SEPT7 and probably SEPT9. As expected, anillin, an important septin and actin binding protein, was also present in overlapping fractions with SEPT2/7. While tubulin-α distribution was used as a control to verify the capability of our approach to analyze the higher order complexes of filamentous proteins, p38 MAPK-distribution is shown as an example of a globular protein predominantly existing as a monomer or in dimeric p38/MK2 complexes smaller than 100 kDa^31^. The wild-type SEPT7 rescued cell line showed similar distribution of all the analyzed proteins to that seen in the floxed line suggesting faithful reproduction of the endogenous septin organization in the dox-induced rescue system. In all the other cell lines analyzed, SEPT7 eluted as oligomeric species in fractions corresponding to molecular weights in the range of ~300–900 KDa, except for S63N mutant which eluted majorly in the range of ~200–440 KDa oligomers (Fig. 8, right panel). Interestingly, the S63N mutant cell line showed significant distribution differences also for the other septins tested, suggesting significant alterations in the hetero-oligomer assembly. Surprisingly and despite the differences observed in filament organization by immunofluorescence, gel filtration data did not reveal any significant differences in cells rescued by the other mutants including the monomeric E202A mutant. Thus, while the homotypic SEPT7 G-interface interaction seems to be dispensable for the survival and proliferation of fibroblasts, the heteropolymeric assembly and higher order filament formation of SEPT7 could involve complex GTP-binding and hydrolysis cycles.

## Discussion

**Figure 9:**
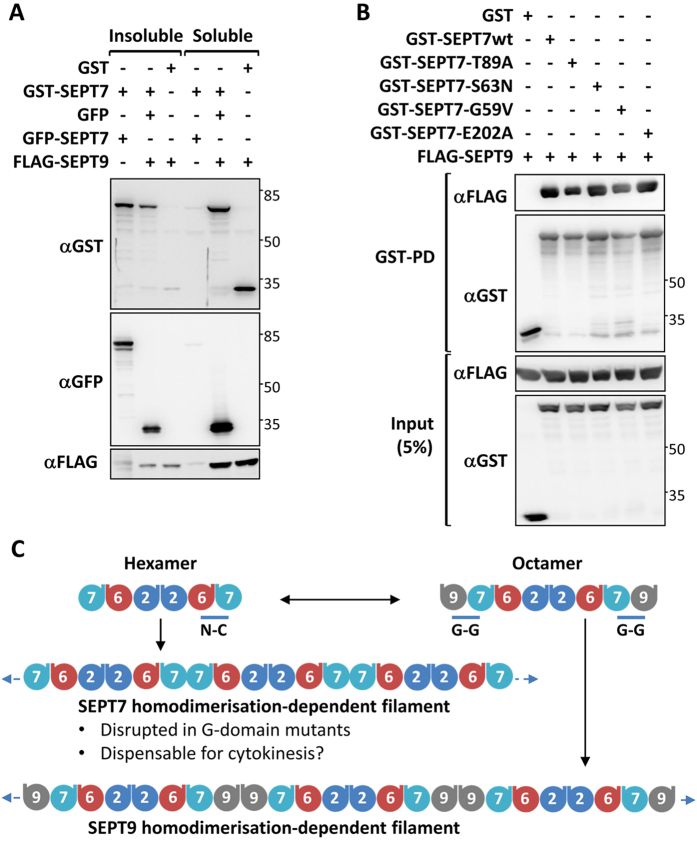
SEPT7-SEPT9 hetero-dimerization is not abrogated by the G-domain mutations of SEPT7. (**A**) The indicate plasmids were co-transfected in HEK293T cells and cells were fractionated into 1% triton soluble and insoluble fractions and equal volumes of lysates were analyzed by western blotting and probed with indicated antibodies. (**B**) GST-tagged wild-type SEPT7 and G-domain mutants were cotransfected with FLAG-SEPT9 expression vector and lysates were subjected to GST-pulldown (PD). The pulldown sampled and input control lysates were analyzed by immunoblotting. (**C**) Scheme for the organization of a septin hetero-oligomeric complexes depicting SEPT9 dependent (octamer) and independent (hexamer) oligomers and polymers.

So far, *in vitro* approaches were used to characterize the filament formation and GTP-binding activities of mammalian septins^7^,^24^. These studies helped in gaining important insights into the rules governing the septin heteropolymer assembly and the significance of the GTPase domain interfaces in maintaining subunit interactions. The fibroblast rescue model used here opens the possibility to conclusively score the effects of mutations in SEPT7 in a process, where septins are absolutely required. We utilized this model to understand the role played by the well-characterized G-G interface of SEPT7 in fibroblast cytokinesis. The observation that the SEPT7 mutations, despite their alterations in filament assembly and oligomerization (S63N), could rescue the proliferation defects of the SEPT7 knockout fibroblasts indicates that the native-like filament assembly of septins could be dispensable for cytokinesis. Electron microscopic studies in the budding yeast have shown that not the presence of any individual septin isoform, but the eventual polymerized filaments at the bud neck is critical for effective budding^17^,^18^. Cdc10 deficient yeasts were viable and formed septin filaments *in vitro* and at the bud neck, but the filaments were of altered morphology as with the mutants in the current study. In a similar observation, SEPT9 deficient fibroblasts were found to have shorter SEPT7 filaments, but could proliferate normally^20^. Apparent lack of any effect of the monomeric E202A mutant challenges the importance of SEPT7-SEPT7 dimerization interface in the formation of functional septin structures in cytokinesis. Previous studies have established the presence of both hexameric (SEPT7:6:2:2:6:7) and octameric (SEPT9:7:6:2:2:6:7:9) heterocomplexes in mammalian cells with many cell-types showing predominant presence of octamers^8^,^32^. While the polymerization of the hexamers involves G-domain driven SEPT7-homodimerization, the octamer- polymerization depends on homotypic SEPT9 interactions via their N-C interfaces^8^. Interestingly co-expression of SEPT9 greatly enhanced the solubility of SEPT7 homopolymers in HEK293T cells, showing that the SEPT7-SEPT9 interactions could interfere with SEPT7-SEPT7 interactions (Fig. 9A). In addition, pulldown assays could show that none of the G-domain mutations interfered with the G-G interface mediated SEPT7-SEPT9 interaction (Fig. 9B). This suggests that the octamers or polymerized octamers are critical for cytokinesis and the altered morphology of filaments in mutants could arise from the lack of the hexamer-driven filaments which are functionally dispensable for cytokinesis (Fig. 9C). SEPT9 has been shown to mediate the final stages of cytokinetic abscission by recruiting the exocyst complex to the midbody and the recruitment of SEPT9 to the abscission site could be the major physiological function of SEPT7 in cytokinesis^33^,^34^.

The results from the real-time GTPase assays demonstrate the complex interplay of GTP-hydrolysis with SEPT7 dimerization due to the fact that SEPT7-dimerization could cause a steric blockade for continuous guanine nucleotide dissociation and reloading (Supplementary Fig. 3). This also makes it hard to perform reliable GTPase measurements and necessitated the use of a highly sensitive indirect assay used in our study. The effects seen with the mutants generated here could be explained based on differences in dimerization strength and affinity for guanine nucleotides. A reduced nucleotide binding affinity could result in enhanced GTP-hydrolysis rates for the G59V and E202A mutants due to enhanced dissociation rates of GDP. Considering the diffused distribution and solubility profile of these mutants upon over-expression, it could be speculated that the enhanced GTPase activity rates are due to better accessibility of the nucleotide binding domains of these proteins due to the lack of homodimerization via the G-G interface. Consistent with this assumption, S63N and T89A mutations, which show only minor effects in the homopolymerization assay, show GTP-hydrolysis rates similar to that of the wild-type GTPase domain. Although these mutants behave similar to the wild-type GTPase domain in the assay, it still needs to be verified whether their reduced activity is exclusively due to reduced GDP dissociation due to G-G dimerization or due to lack of hydrolysis. Although the S63N mutation was expected to have reduced nucleotide affinity, we did not observe any detectable difference for this mutant with respect to nucleotide binding even though there was a moderate increase in the GTPase activity. The T89A mutant could be still associated with GTP in the dimer as observed for the SEPT6 family members, due to the absence of the catalytic threonine in the switch-I region. This hypothesis is well supported by our mass-spectrometric analysis showing enhanced GTP binding on the T89A mutant. Double mutants should be generated by combining the E202A mutation with the other GTPase domain mutants to assess the effects on intrinsic GTPase activity without the interference of dimerization. In addition, similar studies need to be performed with full length SEPT7 alone and in the trimeric complex to make some conclusions on what happens *in vivo*. The negative influence of dimerization on GTPase activity of SEPT7 suggests the active requirement for additional cellular factors for activating the septin GTPase for efficient GTP turnover. Recent studies have identified the possibility of Orc6 acting as a GTPase activating protein (GAP) for septins in Drosophila^33^,^35^, but no septin specific guanine nucleotide exchange factors (GEFs) or GAPs have been identified in mammalian cells. The parent mutations on Ras, which were used as the basis for SEPT7-S63N and -G59V mutants, were shown to affect the Ras GEF- and GAP-activity respectively^36^,^37^. It needs to be answered whether there are specific GAPs/GEFs which could enhance the SEPT7-GTPase activity *in vivo* and which could also help us to understand the mutants better.

Studies on yeast Cdc10/Cdc12 mutants have conclusively demonstrated a requirement for guanine nucleotide binding in septin collar formation, but GTPase activity mutants did not display any defect^22^. The SEPT7-S63N mutant was designed to be deficient in GTP binding and, interestingly, the gel filtration data show that this mutant displays irregularities in hetero-oligomerization. In the homopolymerization assay, this mutant showed the presence of strong filaments together with a large amount of diffusely distributed GFP-tagged protein. Is it possible that the S63N mutant requires higher septin concentrations to polymerize? The heteropolymer assembly of yeast septins in the absence of Cdc10 was shown to require higher septin and GTP concentrations *in vitro*. In that case, *in vitro* dissociation of the hetero-oligomers could be the reason for the absence of higher molecular weight oligomers in gel filtration analysis. This could highlight a unique biochemical property of this mutant and also explains the presence of higher order filaments observed by immunofluorescence. A comprehensive analysis of filament disrupting septin mutations in yeast concluded that the majority of the mutations are at the G-G interface^4^. A more interesting aspect was that most of these mutations gave temperature sensitive phenotypes^4^,^38^. These mutants include the mutations of the G1 box like SEPT7-G59V. Is it possible that the septin G-G interface integrity as well as GTP-binding/hydrolysis activity is important only at higher temperatures or other stress conditions? Future studies using the mutant SEPT7 rescued lines under stress conditions could answer this question. These mutant lines could be of great help in monitoring the role of G-domain interactions in other non-canonical functions of septins in endocytosis, cell migration and bacterial pathogenesis.

The predominant monomeric distribution of SEPT6 observed in the gel-filtration analysis challenges the established models of septin oligomer formation wherein SEPT6 acts as a bridge in mediating SEPT2-SEPT7 interaction. The presence of non-canonical SEPT2/7/9 filaments in fibroblasts cannot be ruled out, but is rather unlikely based on currently available biochemical information and interaction data^6^,^39^. While SEPT6 seems to be strongly expressed in fibroblasts, it cannot be ruled out that another SEPT6 family member (SEPT8/10/11/14) is involved in septin hetero-polymer assembly in these cells. SEPT8 protein could not be detected in these cells, but *Sept8*/*Sept10* transcripts were readily detectable by RT-qPCR (data not shown). Further analysis is required to completely characterize the full spectrum of septin isoforms expressed in order to propose possible alternate models for septin oligomerization.

## Materials and Methods

### Antibodies and Reagents

Anti-SEPT7 antibodies were obtained from IBL international (#JP18991), rabbit anti-anillin antibodies were reported earlier^40^. Other antibodies used were SEPT2 (#11397-1-AP, Acris), SEPT9 (#10769-1-AP, Acris), SEPT6 (sc-20180, Santa Cruz Biotech), SEPT8 (sc-48937, Santa Cruz Biotech), EF2 (sc-13004-R, Santa Cruz Biotech), GAPDH (#MAB374, Millipore), tubulin-α (T6199, Sigma), GFP (sc-9996, Santa Cruz Biotech), GST (sc-138, Santa Cruz Biotech), FLAG (#200472-21, Stratagene) and p38 MAPK (#9212, Cell Signaling Technology). All Alexa-dye labeled secondary antibodies and Alexa fluor-647-conjugated phalloidin (#A22287) were from Invitrogen. HRP-labeled anti-mouse (#115-035-003) and anti-rabbit (#111-035-003) secondary antibodies were from Dianova; anti-goat secondary antibody was from Santa Cruz Biotech (sc-2033). DAPI for DNA staining was from Carl Roth (#6335.1). Polybrene (H9268), doxycycline (D9891) were from Sigma.

### Cloning and mutagenesis

For all cloning and expression studies mouse *Sept7 transcript variant 2* (NM_001205367.1) cDNA was used. *Sept7* cDNA was cloned into the *pSERS-T11-MCS-IRES-EGFP-M2* vector backbone using the In-fusion cloning kit protocol (Clontech). For cloning GFP-tagged SEPT7, PCR amplified *Sept7* CDS was sub-cloned into *Hind*III/*BamH*I double-digested *pEGFP-C3* vector (Clontech). *pDEST27-SEPT7* (GST-tagged SEPT7) was generated by gateway cloning and FLAG-tagged SEPT9 expression construct was described previously^41^. For recombinant protein expression, a *Sept7* cDNA fragment encoding amino acid residues 47–315 (mouse SEPT7 isoform 2, NP_001192296.1) was PCR amplified and inserted into *BamH*I/*Not*I digested *pGEX-4T1-TEV* vector (*pGEX-4T1* with a TEV protease cleavage site). All mutations were introduced using the Quik change-II site directed mutagenesis kit protocol (Agilent).

### Cell culture protocols

All cell lines were maintained in standard growth medium -DMEM/high glucose, 1 mM glutamine, 10% FCS (20% for MEFs), 1x Penicillin/Streptomycin. Transient transfections were performed using polyethyleneimine (PEI) reagent as described previously^24^. *Sept7* floxed mouse (*Sept7*^*tm1Mgl*^)^14^ embryonic fibroblasts were generated from E14 day embryos. Aseptically minced embryos after removing head and red organs were further disaggregated by mincing in a solution of Trypsin-EDTA/DNAseI and incubation with 5 mm glass beads with vigorous shaking at RT for 15 min. The isolated cells were transferred to a fresh tube and resuspended in standard growth medium with 50 μg/ml gentamycin, plated in T25 flasks and propagated at 37 °C with 5% CO_2_. The cells were splitted 1:4 and maintained in the same growth medium. To immortalize primary MEFs, cells were co-transfected with pSV40Tag encoding simian virus 40 large T-antigen and *pREP8* plasmid (Invitrogen) in a 10:1 mixture; colonies were selected with 2 mM histidinol (Sigma). For proliferation assays, MEF clones were seeded in 96 well plates and on alternate days viable cell population were quantified by WST-1 assay (Roche).

### Retroviral packaging and MEF transduction

Gammaretroviral particles (pRBid–Cre) were packaged as described previously^42^. Doxycycline inducible retroviral expression vector used for generating wild-type and mutant-SEPT7-IRES-EGFP cell lines were packaged as described previously^43^. Immortalized MEFs were seeded in 24 well plates (2.5 × 10^4^ cells/well) day before transduction. Plates with viral particles in the presence of polybrene (8 μg/ml) were spun at 1200 × g for 1 h at 32 °C. After overnight virus treatment, cells were washed, medium changed and processed as indicated. For rescue analysis by FACS, the cells were initially transduced with the septin expression vectors, were sorted and retransduced with pRbid-Cre particles.

### Flow cytometric analysis of rescued cells

MEF cells were trypsinized, resuspended in growth medium and analysed by Accuri-C6 flow cytometer (BD Biosciences) under standard settings for detecting GFP (FL-1) and mCherry signals (FL-3) using a 488 nm laser. A MoFlo XDP cell sorter (Beckman-Coulter) was used for sorting mCherry-GFP double-positive cells.

### DNA isolation and genotyping

DNA was isolated from MEFs by over-night lysis at 53 °C in DNA extraction buffer (50 mM Tris-Cl (pH 8.0), 100 mM EDTA, 100 mM NaCl, 1% SDS and 0.5 mg/ml proteinase-K) followed by isopropanol precipitation. Genotyping for *Sept7* was performed using a 3-primer protocol as described previously^14^. PCR reactions were separated on 2% agarose gels and images acquired using INTAS Gel documentation system.

### Assay for solubility of over-expressed GFP-SEPT7

Transfected HEK293T cells were lysed with solubility lysis buffer (20 mM Tris-Cl (pH 7.0), 100 μM EDTA, 1 mM EGTA, 1 mM Sodium ortho-vanadate, 10 mM β-glycerophosphate, 50 mM sodium fluoride, 1% Triton X-100, protease and phosphatase inhibitors) on ice and cleared by centrifugation at maximum speed for 15 min. The supernatant was collected and diluted 1:1 with 4x gel loading buffer. The pellet was washed with lysis buffer, pelleted as before and lysed in 2x gel loading buffer (twice the volume used for cell lysis). All samples were boiled and equal volumes of soluble and pellet samples were analyzed by SDS-PAGE and western blotting.

### Microscopy and immunofluorescence staining

Bright field and SYTOX green (Invitrogen) stained fluorescent images of cells growing in culture plates were acquired using a Leica DM IL LED microscope and Leica EC3 camera. Cells were fixed with cold (−20 °C) ethanol prior to staining with 500 nM SYSTOX green solution in PBS. Fluorescent and bright field images were merged and the percentage of multinucleation was calculated using Image J software (NIH- http://rsb.info.nih.gov/ij/). For Immunofluorescence staining, MEFs were grown on glass coverslips and fixed with 4% paraformaldehyde (PFA) in PBS. Fixation was performed for 2–5 min at room temperature (RT) followed by 20 min at 4 °C and quenching. Cells were permeabilized with 0.25% Triton X-100 in PBS for 30 min at RT. Blocking was done using 4% bovine serum albumin (BSA) for 1 h at 4 °C. Primary antibodies were used at a 1:100 to 1:200 dilution in 1% BSA in PBS for 1–2 h. Secondary antibodies or Alexa Fluor 647-conjugated phalloidin were used at a 1:500 dilution in 1% BSA in PBS. For live-cell imaging, transfected HEK293T cells were seeded in chamber slides one day before analysis. Cells were shifted to CO_2_-independent culture medium and GFP-fluorescence and DIC images were acquired. Imaging was performed using a Leica TCS SP2 confocal microscope with standard settings.

### Size-exclusion chromatography

15 cm plates each of doxycycline-treated rescue clones were lysed in 600 μl of the solubility lysis buffer and incubated on ice for 30 min. The lysate was spun at 2.000 rpm at 4 °C for 5 min to remove the nuclear fraction and further clarified by ultracentrifugation at 80.000 rpm (~250000 × g) at 4 °C for 40 min (Beckmann, TLA 100.4 rotor). 200 μl of the supernatant obtained was loaded onto a Superose6 10/30 column and the proteins were eluted as 800 μl fractions in a buffer containing 20 mM Tris pH8.0 and 100 mM NaCl using an ÄKTA purifier. Aliquotes of the fractions were mixed with equal volumes of 4x SDS-sample buffer and were subjected to SDS-PAGE and western immuno-blotting with indicated antibodies.

### GST-Pulldown analysis

Transfected HEK293T cells were lysed in the solubility lysis buffer with vigorous shaking at 4 °C for 30–45 min. GST-pulldowns were performed by overnight incubation with glutathione beads as described previously^24^.

### Expression and purification of of SEPT7-GTPase domains

*E. coli* BL21 (DE3) strain was used for expressing and purifying proteins from *pGEX-4T1-TEV-SEPT7*_*47–315*_ vectors encoding wild-type and mutant SEPT7. Cultures were grown to an OD600 of ~0.8 and expression was induced with 0.4 mM IPTG at 18 °C overnight. For GST-SEPT7_47–315_ purification, the cells were lysed in buffer A (50 mM HEPES (pH7.5), 500 mM NaCl, 5 mM MgCl_2_, 2 mM DTE) supplemented with 10% glycerol, 5 mM benzamidine, 1 μg/ml pepstatin, 1% NP-40, 1 mM PMSF and 0.2 mg/ml lysozyme. The supernatant obtained upon lysate clarification as stated above, was loaded onto the GST beads equilibrated with buffer A. To obtain GST-free SEPT7_47–315_, the beads washed with 80-100 bed volumes of buffer A and subjected to on-column TEV protease digestion in buffer B (50 mM HEPES (pH7.5), 300 mM NaCl, 5 mM MgCl2, 10% glycerol, 5% glucose, 2 mM DTE) at 4 °C. The SEPT7 containing flow-through was concentrated further and stored at −80 °C following flash-freezing.

### SDS-PAGE and Immunoblotting

Cells were lysed directly in SDS gel loading dye and western blotting was performed as previously described using gradient SDS-PAGE gels^44^. Protein band intensities were quantified using Image J software (NIH- http://rsb.info.nih.gov/ij/). Recombinant proteins were separated on SDS-PAGE, fixed and stained with Coomassie blue microwave protocol and visualized by Fujifilm-LAS3000 imager.

### GTP-association and hydrolysis assays

For mass spectrometric analysis of GTP/GDP-association, tag-free SEPT7-GTPase domains in buffer B was boiled to 95 °C for 1 min and cleared by centrifugation. The supernatants collected after centrifugation were analysed by HPLC-coupled tandem mass spectrometry in comparison to authentic GTP and GDP standards. Regenerative coupled-enzyme based GTPase assay^29^ was performed with 20 μM of SEPT7 (47–315) protein. For a total reaction volume of 50 μl, GTP was added to a final concentration of 1 mM in the reaction mix containing SEPT7 (47–315), 0.2 mM NADH, 0.5 mM PEP, 0.02 mg/ml Lactate dehydrogenase and 0.05 mg/ml pyruvate kinase in buffer C. The reaction was monitored (absorbance at 340 nm) continuously for 2 h at intervals of 20 sec at 25 °C in a 96-well format using Thermo Scientific Multiskan FC Microplate photometer. The rate of GTP hydrolysis was calculated as: rate of GTP hydrolysis (sec^−1^) = −ΔA_340_/(ε c l), where −ΔA_340_ refers to the change in absorbance per sec at 340 nm, ε refers to the molar extinction coefficient of NADH at 340 nm (6220 M^−1^cm^−1^), l refers to the path length and c refers to the protein concentration.

## Additional Information

**How to cite this article**: Abbey, M. *et al*. GTPase domain driven dimerization of SEPT7 is dispensable for the critical role of septins in fibroblast cytokinesis. *Sci. Rep.* **6**, 20007; doi: 10.1038/srep20007 (2016).

## Supplementary Material

Supplementary Information
