# Supplementary material for: GTPase domain driven dimerization of SEPT7 is dispensable for the critical role of septins in fibroblast cytokinesis
Source: Sci Rep. 2016 Jan 28;6:20007. doi: 10.1038/srep20007 (PMC4730212; doi:10.1038/srep20007)
Supplement: Supplementary Information [file srep20007-s1.pdf]

Supplementary data
(Supplementary Figures 1 - 3)

GTPase domain driven dimerization of SEPT7 is dispensable for the critical role of septins in fibroblast cytokinesis

Megha Abbey[1], Cosima Hakim[1], Roopsee Anand[2], Juri Lafera[1], Axel Schambach[3], Andreas Kispert[4], Manuel H. Taft[2], Volkhard Kaever[5, 6], Alexey Kotlyarov[1], Matthias Gaestel[1*], and Manoj B. Menon[1*]

Institute of Physiological Chemistry[1], Institute of Biophysical Chemistry[2], Institute of Experimental Hematology[3], Institute of Molecular Biology[4], Institute of Pharmacology[5] & Research Core Unit Metabolomics[6], Hannover Medical School, Hannover- 30625, Germany.

Supplementary Figure 1

A

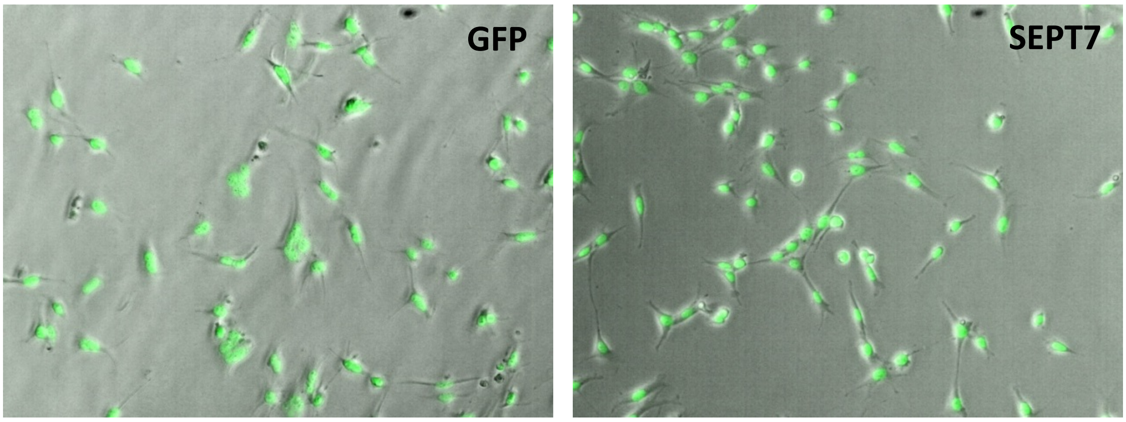

B

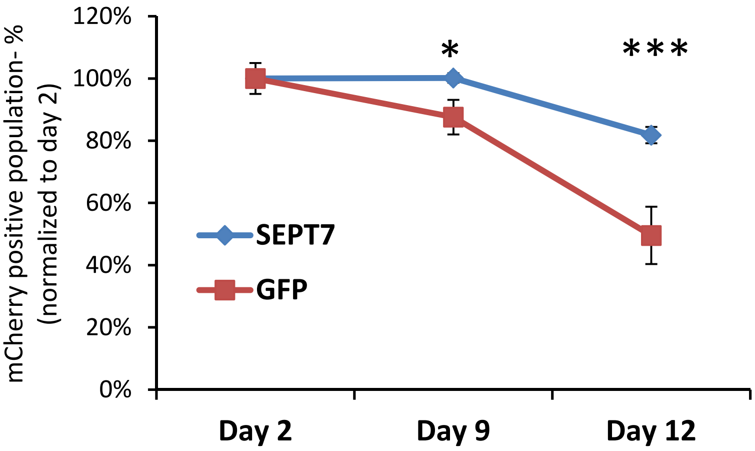

Supplementary Figure 1. Verification of the Dox-inducible rescue model.

A. Representative images for data presented in Figure 1J. GFP or SEPT7-IRES GFP expressing cells were transduced with pRBid-Cre and cells were sorted 3 days later. The sorted GFP/mCherry double positive cells were fixed 3 days later and stained and imaged . 4x wells of each genotype were analyzed . **B.** In a parallel experiment, the loss of mCherry transduced cells were monitored in GFP or SEPT7 expressing cells by flow-cytometry (+Dox + Cre) as in Figure 1H. Triplicate analysis represented +/- SD shows the robustness of the assay (* denotes a p value of 0.0505 & *** denotes a p value of 0.00055; t test, n= 3).

Supplementary Figure 2

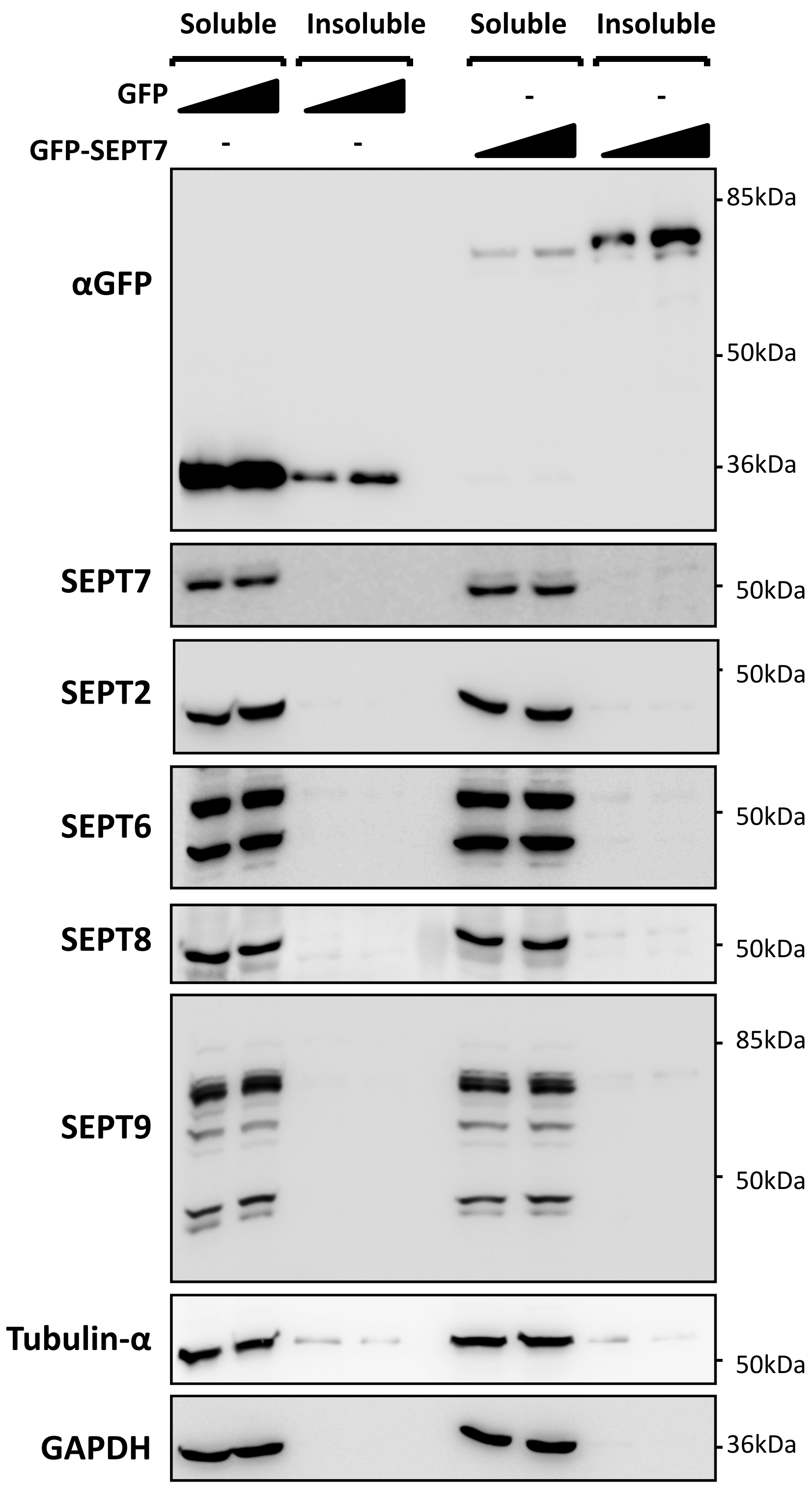

Supplementary Figure 2. Over expressed GFP-SEPT7 forms insoluble homopolymers in HEK 293T cells. GPP / GFP-SEPT7 transfected HEK 293T cells were fractionated into 1% triton soluble and insoluble fractions and equal volumes of lysates were analyzed by western blotting and probed with indicated antibodies. While GFP-SEPT7 is predominantly present in the insoluble fraction, GFP and all the other endogenous septins are in soluble fractions. GFP-SEPT7 has no effect on the solubility profile of endogenous septins, suggesting that the insoluble filaments are homo-polymeric and do not contain endogenous septins.

Supplementary Figure 3

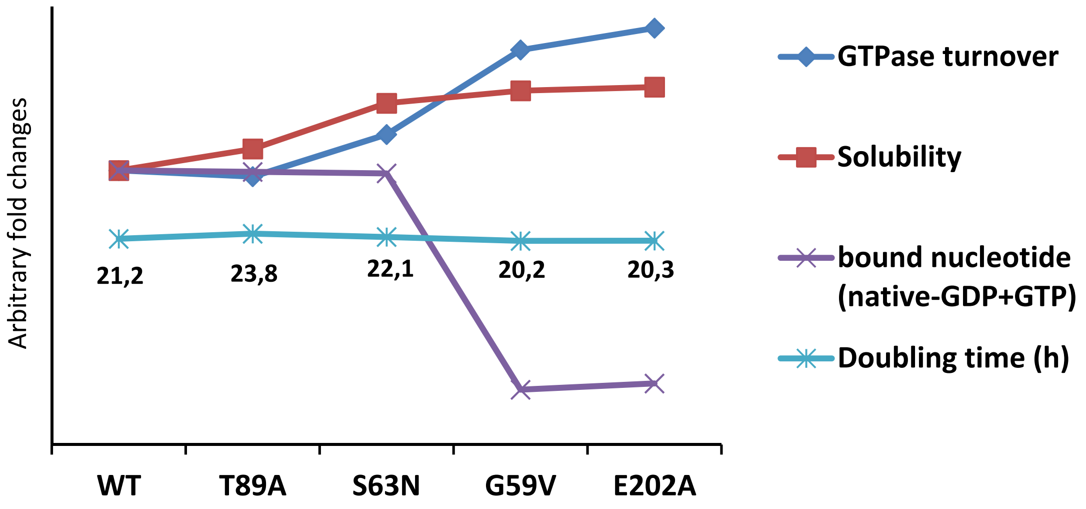

Supplementary Figure 3. Effect of G-domain mutations on multiple parameters analyzed in the study. When the *in-vitro* GTPase turnover (Fig. 4C) and nucleotide-binding (Fig. 4B) data is coupled to cell-based solubility assays (Fig. 3) and doubling time (Fig. 6E) of the rescued clones- there is a positive correlation between doubling time and nucleotide-binding and an inverse correlation between GTPase turnover/solubility versus nucleotide binding.
